# The Role of Noninvasive Respiratory Management in Patients with Severe COVID-19 Pneumonia

**DOI:** 10.3390/jpm11090884

**Published:** 2021-09-03

**Authors:** Evangelia Akoumianaki, Eleni Ischaki, Konstantinos Karagiannis, Ioanna Sigala, Spyros Zakyn-thinos

**Affiliations:** 1 Department of Intensive Care Unit, University Hospital of Heraklion, 71500 Crete, Greece; 2 First Department of Intensive Care Medicine, National and Kapodistrian University of Athens Medical School, Evaggelismos General Hospital, 10676 Athens, Greece; eischaki@yahoo.gr (E.I.); giannasig@yahoo.com (I.S.); szakynthinos@yahoo.com (S.Z.-t.); 3 Department of Respiratory Medicine, University Hospital of Heraklion, 71500 Crete, Greece; k.karagiannis@med.uoc.gr; 4 School of Medicine, National and Kapodistrian University of Athens, 10676 Athens, Greece

**Keywords:** COVID-19, noninvasive respiratory treatment, High Flow Nasal Oxygen, Noninvasive Ventilation, SARS-COV-2

## Abstract

Acute hypoxemic respiratory failure is the principal cause of hospitalization, invasive mechanical ventilation and death in severe COVID-19 infection. Nearly half of intubated patients with COVID-19 eventually die. High-Flow Nasal Oxygen (HFNO) and Noninvasive Ventilation (NIV) constitute valuable tools to avert endotracheal intubation in patients with severe COVID-19 pneumonia who do not respond to conventional oxygen treatment. Sparing Intensive Care Unit beds and reducing intubation-related complications may save lives in the pandemic era. The main drawback of HFNO and/or NIV is intubation delay. Cautious selection of patients with severe hypoxemia due to COVID-19 disease, close monitoring and appropriate employment and titration of HFNO and/or NIV can increase the rate of success and eliminate the risk of intubation delay. At the same time, all precautions to protect the healthcare personnel from viral transmission should be taken. In this review, we summarize the evidence supporting the application of HFNO and NIV in severe COVID-19 hypoxemic respiratory failure, analyse the risks associated with their use and provide a path for their proper implementation.

## 1. Introduction

During the last year, SARS-COV-2 has rapidly spread worldwide, causing millions of deaths. Most hospitalized patients present with hypoxemic acute respiratory failure (ARF) and a small proportion of them require admission to the intensive care unit (ICU). The mortality in patients who require invasive mechanical ventilation (MV) due to severe COVID-19 pneumonia is around 40%. The high mortality rate along with the shortage of ICU beds render the avoidance of intubation, when feasible, essential for prognosis [1]. In this context, noninvasive respiratory treatment modalities, such as High-Flow Nasal Oxygen and Noninvasive Ventilation, have been widely adopted in patients with hypoxemic ARF secondary to COVID-19. High-Flow Nasal Oxygen (HFNO) is a noninvasive respiratory support modality that delivers warm, humidified oxygen at a maximum flow rate of 60 L/min and up to 100% of the inspired oxygen fraction (FiO_2_) through nasal probes [2,3]. In comparison to conventional oxygen treatment where the flow rates are low (< 15 L/min), the high flow rates of HFNO more adequately meet the increased inspiratory demands of patients with respiratory distress. Furthermore, high-flow inspiratory rates minimize the entrainment of room air and ensure a higher and more precise FiO_2_. Additional pathophysiologic benefits include the generation of low positive end-expiratory pressure (PEEP), allowing the recruitment of alveolar units and the reduction of dead space by washing out carbon dioxide from the upper airways [3,4]. Noninvasive Ventilation (NIV) refers to the application of mechanical ventilatory support using a nasal, oronasal, or full-face mask, or a helmet [5]. Its beneficial physiologic effects consist of hypoxemia improvement and respiratory muscles unloading. The most commonly used NIV modalities are continuous positive airway pressure (CPAP) and bilevel positive airway pressure (BiPAP). Although there is substantial controversy as to whether CPAP can be considered as NIV, in this document NIV will be used to refer to both BiPAP and CPAP if not otherwise specified. The use of BiPAP has become the standard of care in patients with hypercapnic respiratory failure due to acute exacerbation of chronic obstructive pulmonary disease (AECOPD), while NIV (CPAP or BiPAP) is considered a treatment option for acute respiratory failure due to cardiogenic pulmonary oedema (CPO) [6,7]. Nevertheless, the use of NIV for hypoxemic ARF without prior chronic respiratory disease (de novo ARF), which represents the most common life-threatening complication of COVID-19, remains debatable [7].

This is a narrative review that aims to analyse all available evidence on the effectiveness and risks of NIV and HFNO in severe COVID-19 disease, and to provide a practical path for their safe application in this group of patients. We searched two databases, PubMed and Google Scholar, using the following search terms: “high flow oxygen AND COVID-19”, “high flow oxygen AND SARS-CoV 2”, “noninvasive ventilation AND COVID-19”, “noninvasive ventilation AND SARS-CoV 2” and “noninvasive respiratory management AND COVID-19”. All types of articles related to humans were included. Articles for which full text was not available, were not in English, or were not published in PubMed were excluded. From the articles retrieved in the first round of searching, additional references were identified by a manual search among the cited references.

## 2. High-Flow Nasal Oxygen in Patients with COVID-19-Associated Respiratory Failure: Evidence for Potential Benefit

There are two main outcomes of interest when assessing the value of HFNO in patients with respiratory failure associated with COVID-19: the impact of treatment on endotracheal intubation and mortality. The available evidence indicates that HFNO prevents intubation in a considerable number of patients with severe COVID19, but, so far, this has not been clearly associated with a survival benefit. 

Worldwide, a proportion of 23–64% of patients with severe COVID-19 pneumonia has received HFNO [8,9,10,11,12,13,14]. This practice was based on the evidence originating from the era prior to SARS-Cov-2, which indicated that HFNO significantly reduced the need for endotracheal intubation in most patients with severe acute hypoxemic respiratory failure [15,16,17,18,19,20,21,22,23,24]. However, with the exception of one study [2], no apparent effect of HFNO on mortality or ICU length of stay has been indicated [15,16,20,21,22,23,24,25,26]. In severe hypoxemia associated with COVID-19, the available data of HFNO performance come almost exclusively from retrospective observational cohorts (Table 1). HFNO managed to avert escalation of treatment and/or intubation in approximately half of the patients with COVID-19-related hypoxemia (44 to 64%) [8,9,10,11,27,28,29,30]. The very few studies that compared HFNO to standard oxygen therapy found that HFNO reduced intubation and subsequent invasive MV without affecting ICU length of stay or mortality [10,11,31]. Demoule et al. matched 137 patients with severe COVID-19 who received HFNO with 137 patients who did not [11]. Significantly less patients were intubated by Day 28 in the HFNO group (55% vs. 72%, *p* < 0.0001). Mortality was the same between the two groups (21% in the HFNO group vs. 22% in the other). It should be noted that the studies that assessed the effect of HFNO in severe COVID-19 pneumonia were retrospective in nature and underpowered to detect a meaningful difference in mortality. We need properly designed, large-scale trials answering whether HFNO affects mortality in severe COVID-19, both directly but also indirectly, by reducing the ICU-related complications and increasing the availability of ICU beds through endotracheal intubation decrease.

## 3. Noninvasive Ventilation in Patients with COVID-19-Associated Respiratory Failure: Evidence for Potential Benefit

The use of NIV in COVID-19 has been mostly based on data derived from studies of patients with de novo ARF. Although the results of these studies are conflicting [32,33,34,35,36], two recent meta analyses showed a remarkable reduction in intubation rates and a statistically significant improvement in survival was demonstrated in one of them [37,38]. The main studies that investigated the management of patients with COVID-19 with NIV are presented in Table 2. One of the first observational studies for NIV use in COVID-19 reported no significant differences in 30-day mortality (approximately 30%) and risk of intubation (25–28%) between NIV, CPAP and HFNO therapy in a non-ICU environment [39]. Another large observational study in Germany reported high mortality (53%) in COVID-19 patients who received invasive MV, whereas mortality was lower in the subgroup of patients who received Noninvasive Ventilation alone (45%) [40]. Notwithstanding, in those who presented NIV failure, mortality was as high (50%) as in patients treated with invasive MV [40]. Similar results were shown by a sub-analysis from the HOPE COVID-19 registry, in which more than half of the patients who received NIV survived without the need of intubation [41]. Again, the NIV failure group of patients (16%) exhibited increased mortality compared to success (58% in hospital death rate) [41]. It is noteworthy that the majority of COVID-19 patients were treated with CPAP rather than BiPAP in these trials. Additionally, there are quite a few small-scale observational studies that suggest benefit from use of CPAP in COVID 19 hypoxemic ARF [42,43,44,45,46]. Further large-scale trials are necessary in order to identify the group of COVID-19 patients in which NIV use is more beneficial. 

## 4. Risks of Noninvasive Respiratory Treatments in Severe COVID-19

The greatest concerns when applying noninvasive respiratory management in hypoxemic respiratory failure related to COVID-19 are the risk of delaying intubation and the spread of the virus to and among healthcare personnel.

It has been demonstrated that a substantial proportion of patients with hypoxemic ARF do not avoid invasive MV despite NIV or HFNO trials and that this happens more frequently in those with severe hypoxemia [44]. In critically ill patients with COVID-19, Menga et al. found that noninvasive oxygenation strategies were more likely to fail, compared to those with hypoxemic respiratory failure from other reasons [57]. The major concern is that NIV or HFNO failure may adversely affect the outcome, as suggested in several studies [44,58,59,60]. It seems unlikely that noninvasive respiratory management per se increases mortality in patients with severe hypoxemia. Indeed, data coming from very few, mostly observational studies indicate that, neither time from ICU admission to intubation nor noninvasive respiratory therapy adversely affected the outcome of these patients [28,29,61]. The reasonable explanation between HFNO or NIV failure and worse outcome is intubation delay and lung injury worsening. Noninvasive respiratory support cannot guarantee lung protective ventilation [62], as severely hypoxemic patients usually exhibit high respiratory drive and vigorous efforts that enhance lung injury through tidal volume increase, the pendelluft phenomenon, capillary leak and lung oedema [63,64,65,66,67]. In several studies, the magnitude of inspiratory efforts and expired tidal volumes following NIV implementation accurately predicted NIV failure and have been correlated with worsening lung injury and mortality [44,62,68,69]. Furthermore, insufficient unloading of the respiratory muscles during NIV or HFNO may harm the diaphragm and ultimately cause fatigue and respiratory arrest [70]. Therefore, when a patient with severe hypoxemia due to COVID-19 pneumonia is managed with HFNO or NIV, close monitoring of respiratory distress and continuous evaluation of predictors of failure is essential.

Both HFNO and NIV are considered aerosol-generating procedures and, as such, may spread the virus into the environment. There is extensive discussion as to whether management of patients with COVID-19 with noninvasive respiratory modalities increases the risk of healthcare personnel contamination. The available literature on this field comes mostly from simulation or observational studies conducted prior to the COVID-19 pandemic. Results are mixed and inconclusive [21]. A few observational studies described viral hospital spread to HFNO and/or NIV use [39,71,72]. Environmental contamination was higher in patients treated with these modalities compared to MV with closed suction systems [73]. During a cough-simulating scenario, HFNO moderately increased the distance of droplet dispersion by an average of 0.42 m [74]. On the other hand, other investigators demonstrated that there was no significant difference in the risk of aerosol production and dispersion between spontaneous breathing, conventional oxygen treatment and HFNO or NIV therapy [75,76,77,78,79,80,81], and that these modalities do not expose healthcare workers to higher infection risk provided that they follow the appropriate personal protection precautions [82,83,84]. In a very interesting recent article by Gaeckle and coworkers, aerosol generation from healthy participants receiving oxygen via a non-humidified nasal cannula, face mask, HFNO and NIV was measured during normal breathing, talking, deep breathing and coughing [75]. NIV and HFNO did not produce a higher aerosol concentration when compared with breathing room air or non-humidified oxygen modalities [75]. Factors such as higher flow rates during HFNO, augmented positive pressures during NIV and, more importantly, poor fit of these devices to a patient´s nose or mouth carry a higher risk of viral transmission than the oxygen modality used [75,85]. In summary, the exact amount to which HFNO or NIV expose healthcare workers to viral transmission is unknown but it does not seem to differ significantly than spontaneous breathing or conventional oxygen treatments. Any hazard can be minimized if the noninvasive oxygen therapy is applied in negative pressure or well-ventilated rooms; staff wears protective equipment, including FFP3 masks; leaks around the devices are eliminated; and positive pressures and flow rates are at the minimum necessary. Notably, the addition of a simple surgical mask over HFNO may further reduce aerosol and droplet dispersion due to the exhaled gas flow and it is a recommended strategy in patients with severe COVID-19 [86]. Leonard et al demonstrated that wearing a surgical mask captured 83.2% of particles between 0.1–100 μm [86] while, in a recent experimental trial, Hamada et al. provided evidence that this strategy almost completely suppressed particle dispersion induced during coughing [87]. 

## 5. HFNO in COVID-19-Associated Respiratory Failure: Practical Aspects

One of the major concerns regarding HFNO use is the optimal place for it to be applied in order to allow a close monitoring of the patient, without increasing the risk of virus transmission among healthcare workers. Ideally, patients receiving supplemental oxygen via HFNO should be hospitalized in the ICU or in a high-dependency unit (HDU) and, preferably, in negative-pressure rooms. However, the COVID-19 pandemic has caused serious resource and bed limitations. Under circumstances where sparing ICU beds is vital, HFNO could be applied in the non-ICU setting, provided that all precautions against viral transmission are carefully followed and the patient is rigorously monitored to avoid any intubation delay.

**Figure 1:**
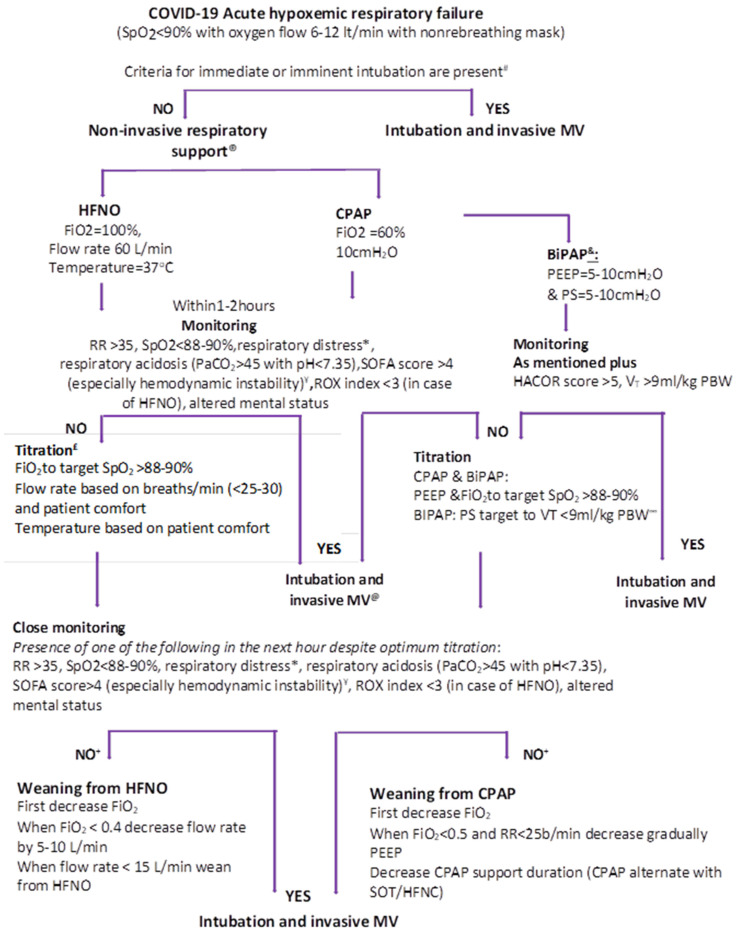
Recommended algorithm for noninvasive respiratory support in COVID-19 patients with acute hypoxemic respiratory failure. Patients with PaCO_2_ > 45 mmHg are excluded. # Criteria for immediate or imminent intubation are impaired consciousness, persistent shock (which is defined by systolic arterial blood pressure < 90 mmHg despite adequate fluid administration), hypercapnia/acidosis and deteriorating respiratory distress. ^®^ The choice between HFNO and NIV depends on device availability and familiarity. In case that both are available, HFNO is proposed as a first choice because of better patient tolerance and ease of use. & BiPAP could be a choice in case of respiratory distress. BiPAP initial pressure settings could be different, depending on the interface used, i.e., with helmet pressures it should be increased by 50%. * Respiratory distress is detected by the presence of persistent auxiliary muscle use and/or thoraco-abdominal asynchrony. £ The rationale of change in HFNO settings is the following: (a) increase in flow rate is expected to decrease the respiratory muscle workload with concomitant decrease in the respiratory rate, dyspnoea, auxiliary muscle use and thoraco-abdominal asynchrony; (b) increase in FiO_2_ causes increase in PaO_2_ and SpO_2_; (c) temperature can be set at 37 °C or lower (31–34 °C) based on the patient’s comfort. ¥ Hemodynamic instability is defined by a heart rate >140 beats/min or change >20% from baseline and/or systolic arterial blood pressure > 180 mmHg, <90 mmHg or decrease >40 mmHg from the baseline. @ In case of HFNO failure, a short trial of NIV could be considered in the ICU/HDU area. ∞ BiPAP use should be as much as possible, ideally continuous. + If the patient’s clinical status and arterial blood gases are progressively improved, we proceed to weaning. BiPAP: Bilevel positive airway pressure; CPAP: continuous positive airway pressure; FiO_2_: fraction of inspired oxygen; HACOR score: Heart rate–pH–Glasgow Coma Scale–PaO_2_/FiO_2_–respiratory rate; HFNO: High-Flow Nasal Oxygen; MV: Mechanical Ventilation; PaO2: arterial partial pressure of oxygen; PaCO_2_: arterial partial pressure of carbon dioxide; PBW: predicted body weight; RR: respiratory rate; ROX index:ratio of SpO_2_/FiO_2_ to the respiratory rate; SpO_2_: pulse oximetry of oxygen; SOT: standard oxygen treatment.

In the vast majority of cases, the first step of respiratory support of a patient with COVID-19-related ARF is the application of a conventional oxygen device, such as a nasal prong, Venturi mask or non-rebreather mask. These measures are sufficient to a large extent. However, when the patient either admits with, or develops signs of acute respiratory distress, physicians should check if the criteria for imminent intubation and invasive MV are met [88] (Figure 1). If there is not any indication for intubation, HFNO should be the first choice in case of mild to moderate respiratory distress and SpO_2_ < 90%, despite a Venturi or non-rebreather mask [89]. The initial HFNO settings must be maximal (100% FiO_2_, flow rate 40–60 L·min^−1^ and temperature 37 °C) [88]. From a practical point of view, starting with the highest flow rate (60 L·min^−1^) seems a reasonable approach. Within 1 to 2 h, the HFNO settings should be titrated based on patients’ respiratory rate (<25–30 per minute), SpO_2_ (92–96%) and comfort [90].

One of the most challenging decisions when dealing with a patient with severe COVID-19 pneumonia is to decide if and when intubation is preferable to noninvasive support. Physicians often rely on parameters that have been associated with HFNO failure, such as a high respiratory rate; SpO_2_ < 88–90% under high flow rates and FiO_2_; use of auxiliary respiratory muscles; thoraco-abdominal asynchrony; hypercapnia (PaCO_2_ > 45 mmHg with pH < 7.35) [88,91]; additional organ dysfunction, as expressed by a SOFA score > 4; and mainly hemodynamic instability and an altered mental status [92]. However, when examined independently, the respiratory rate is a poor and often late marker of evolving respiratory disease [93,94]. Furthermore, patients with COVID-19 pneumonia may present without respiratory distress, despite the existence of severe hypoxemia that necessitates aggressive therapeutic correction, including mechanical ventilatory support [14,94]. Respiratory distress may be absent in patients with respiratory failure when respiratory muscle function is normal and the respiratory system mechanics are relatively preserved, which is often the case in severe COVID-19 pneumonia, especially at its early stages [94,95]. Furthermore, studies have demonstrated that PaO_2_ is a weak stimulus of the respiratory center and dyspnoea may not occur despite severe hypoxemia [65]. Finally, when PaCO_2_ is low, the hypoxic ventilator response is considerably attenuated [96].

Because assessing the response to HFNO is complex, prognostic indexes have been developed based on the combination of several prognostic markers of respiratory failure. The most evaluated is the respiratory rate–oxygenation (ROX) index, which is calculated by the ratio of SpO_2_/FiO_2_ to the respiratory rate [97]. In a multicenter, retrospective study, in patients with COVID-19-associated respiratory failure, the ROX index applied at multiple times intervals after the application of HFNO aided in the identification of those patients that could ultimately be weaned from HFNO, although with different cut-off points (ROX index greater than 3.67 at 12 h after the application of HFNO was an accurate predictor of successful weaning) [29]. Conclusively, the physician should bear in mind the risk factors that have been associated with HFNO failure in COVID-19-associated respiratory failure. These include advanced age; the presence of comorbidities and a high initial SOFA score; a low Glasgow Coma Scale; high lactate, procalcitonin and serum lactate dehydrogenase at ICU admission; use of vasopressors; and a low respiratory rate–oxygenation (ROX) index at several time points following HFNO [10,29,30,57,98]. 

If HFNO fails, the patient should be transferred immediately to an ICU or an HDU and treated with a short NIV trial or immediately intubated and ventilated invasively. Sustaining HFNO for respiratory support in an unresponsive patient can result in undesired respiratory and cardiac complications. Instead of a specific time frame after HFNO initiation as a criterion for early or late intubation, the presence of negative prognostic indices and the inability to reverse them within 1 or 2 h after HFNO titration with maximum settings should be considered as more accurate, given the prolonged illness duration of COVID-19 respiratory failure. Besides, prolonged trials of HFNO in patients with COVID-19 respiratory failure are not associated with poor clinical outcomes [29]. We should always bear in mind that patients with a lower PaO_2_/FiO_2_ ratio are more likely to experience HFNO failure and this group should be ideally treated in an ICU/HDU area [8]. 

If the patient’s clinical status and arterial blood gases progressively improve, HFNO should be weaned gradually by first decreasing FiO_2_ to 40–50%, followed by a stepwise decrease in flow rate by 5–10 L·min^−1^ with intervals based on the patient’s respiratory parameters. If the patient remains stable for 1–2 h with FiO_2_ 40% and a flow rate < 15 L·min^−1^, HFNO can be stopped safely and a venturi mask or low-flow nasal prongs can be applied [88].

## 6. Noninvasive Ventilation in COVID-19-Associated Respiratory Failure: Practical Aspects 

The use of NIV in de novo hypoxemic ARF (non-COVID) has a considerable probability of failure—up to 50% in various studies [44,99]. Current guidelines cannot make recommendations regarding the use of NIV in hypoxemic ARF [7]. However, the LUNG-SAFE study showed that, in every day clinical practice, 15.5% of patients with hypoxemic ARF are treated with NIV as the initial management, irrespective of the severity [44]. 

Considering the high probability of failure, NIV-treated patients are hospitalized in an ICU environment where continuous monitoring can be established. Nevertheless, in the context of the COVID-19 pandemic, there is a huge need to use noninvasive modes of ventilation outside the ICU in order to spare ICU beds. NIV is used either in the mode of CPAP or BiPAP. During the first COVID-19 wave in Italy, a “feasibility study” was conducted in order to establish that noninvasive respiratory support can be successfully applied outside an ICU [39]. Indeed, 670 patients were treated with either NIV, helmet CPAP or HFNO in a respiratory ward or respiratory high dependency unit (HDU) with a nurse:patient ratio up to 1:6 and an intubation rate of 27%, with the mortality rate being 26.9%, a quite favourable outcome [39]. Recently, Ward-COVID, a multicentre study from 31 hospitals in Italy successfully applied CPAP/NIV in 909 patients (10.4% of all patients with respiratory failure) in a ward with an NIV failure rate 37.6% [47]. There are plenty of other studies from different countries describing successful application of CPAP [42,46,48,49,50] or NIV [41,51,52] in a ward, with the vast majority of patients receiving CPAP support. However, the experience of staff in the use of these modalities, the nurse/patient ratio and the intensity of the monitoring probably are not similar in all studies. 

Different studies used different inclusion criteria in order to apply NIV support in COVID-19-related ARF. Our knowledge from hypoxemic ARF in the pre-COVID-19 era suggests that application of NIV in patients with a PaO_2_/FiO_2_ < 150 mmHg carries a considerable risk of failure, with increased mortality compared to invasive MV [44]. In the pandemic era, if patients were unable to maintain oxygen saturation above 92% with conventional oxygen, a trial of NIV was offered. Oxygen supply ranging from 6 L/min up to 15 L/min with a nonrebreathing mask was used as the criterion for initiation of NIV support in different trails [39,46,50]. A PaO_2_/FiO_2_ of 100–200 mmHg, breathing frequency above 30/min, dyspnoea level and use of accessory respiratory muscles were used complementary in some trials [45,52]. However, as a general rule, patients had to be hemodynamically stable to be considered available for NIV support outside ICU. Intubation criteria were variable but generally intubation was considered if there was persistent hypoxemia, worsening or respiratory failure or lack of improvement despite NIV support, PaO_2_/FiO_2_ < 100 mmHg, development of respiratory acidosis–hypercapnia, evidence of ongoing respiratory distress and increased breathing, hemodynamic instability and an altered mental status [43,49,51,52,53].

The vast majority of patients with COVID-19-related ARF received NIV outside the ICU in the form of CPAP. CPAP is easier to apply outside ICU, requires less expertise from personnel compared to BiPAP and can be delivered either with a CPAP valve with venturi flow system or a CPAP device. BiPAP has been preferentially used in the more severe patients with respiratory acidosis, hypercapnia, evidence of increased breathing (respiratory muscle fatigue) as well as those with a history of obstructive pulmonary disease or obesity hypoventilation syndrome [39,51,54]. Most CPAP protocols start with the CPAP set at 10 cmH_2_O to target an SpO_2_ ≥ 90% or PaO_2_ ≥ 60 mmHg and then adjusted according to the SpO_2_, respiratory distress and clinical tolerance [46]. The BiPAP setup is more demanding, as the large pressures used augments air leaks and patient–ventilator synchronization is often an issue. BiPAP is usually initiated with a PEEP range of 5–10 cmH_2_O and a PS of 5–10 cmH_2_O, targeting an expiratory tidal volume below 9 mL/kg predicted body weight [100]. 

The safest way is to deliver NIV with a non-vented mask (full face, oronasal or helmet) that covers the mouth and a dual circuit ventilator with a filter on the expiratory limb. In case of single-limb ventilators, a non-vented mask should be used with an antimicrobial filter placed between the interface and the exhalation port. Any effort to minimize the leaks should be made [100].

Italian guidelines support the use of a helmet interface during the pandemic, as a way to minimize personnel exposure [101]. Helmets have been studied in hypoxemic ARF as a way to increase patient tolerance and consequently the time of continuous NIV application and to apply higher PEEP more effectively, minimizing the leaks issue compared to face masks. A randomized study comparing a helmet with face mask interface in patients with ARDS stopped early because the helmet showed a reduction in intubation rate (18.2% vs. 61.5%, respectively) and in 90-day mortality (34.1% vs. 56.4%) [102]. A helmet facilitated greater PEEP and resulted in greater decrease in the respiratory rate than a face mask [102]. In the COVID-19 pandemic, there is no direct comparison between the various interfaces. Helmet CPAP has been successfully applied either in the ward [39,47,49] or HDU [43]. Helmet BiPAP applied in the ICU environment exhibited a lower intubation rate compared to HFNO [55]. However, as a helmet has a large internal volume (dead space) and high compliance, patient–ventilator asynchronies are a frequent issue in BiPAP mode. When the helmet interface is used, pressures (PEEP and pressure support) should be considerably increased (by 50%), a high flow rate should be used to avoid CO_2_ rebreathing and the pressurization rate should be the shortest to optimize patient–ventilation synchrony [103,104]. Continuous monitoring and fine tuning are needed.

Prediction of NIV failure is the holy grail to avoid intubation delay and increased mortality [41,44]. Till now we have evidence that PaO_2_/FiO_2_ < 150 mmHg, expiratory tidal volume > 9.5 mL/kg PBW and HACOR score > 5 at 1 h of NIV carry a high probability of NIV failure [44,62,68,69]. HACOR incorporates heart rate, pH, Glasgow coma scale, PaO_2_/FiO_2_ and respiratory rate in a composite score [105,106]. The HACOR score has not been tested specifically on COVID-19 patients. Ward-COVID, the largest today multicentre study from Italy, demonstrated NIV failure in 53% of patients with PaO_2_/FiO_2_ < 150 mmHg vs. 18% in patients with PaO_2_/FiO_2_ > 150 mmHg [47]. Consistent with that, Coppadoro et al. showed that PaO_2_/FiO_2_ < 100 mmHg with helmet CPAP was associated with a high probability of failure [49]. Moreover, an increase in oxygenation with CPAP treatment and a decrease in the respiratory rate (<24/min) were strong indices of success [49]. Ahmed et al. demonstrated that the CPAP failure group had a higher respiratory rate, whereas a SpO_2_ to FiO_2_ ratio ≥ 114 pre-CPAP or ≥ 180 at 30–120 min post CPAP could differentiate the success group [56]. However, none of the above can be applied as a general rule predicting NIV failure or success, and individual consideration and close monitoring of the patient are needed.

## 7. High-Flow Nasal Oxygen vs. Noninvasive Ventilation Patients with COVID-19-Associated Respiratory Failure 

The current international guidelines vary widely concerning the optimal noninvasive respiratory support for patients with COVID-19-related hypoxemia, reflecting the lack of large-scale randomized control trials in this field [107,108]. Hence, current clinical practice is based on prior experience, personal medical opinion and local availability.

In non-COVID-19 patients with hypoxemic ARF, the evidence about the ideal noninvasive respiratory support strategy are scarce. Some studies showed a reduced intubation rate with HFNO in more severely hypoxemic patients [2], while others did not demonstrate any benefit from HFNO or NIV with respect to endotracheal intubation or mortality [15,109]. 

Only a few studies performed a head-to-head comparison of NIV to HFNOT in COVID-19. The HENIVOT, an open-label, multicentre randomized controlled trial, compared helmet NIV with HFNO in 110 patients with moderate to severe hypoxemic ARF secondary to COVID-19 pneumonia [55]. At 28 days post randomization, NIV with a helmet did not show any significant difference in days free of respiratory support as compared to HFNO. Nevertheless, the use of a helmet significantly reduced the intubation rate and increased invasion ventilation-free days in comparison to HFNO [55]. A retrospective observational study in patients with COVID-19 did not find differences in the mortality rate between HFNO, CPAP and NIV after adjustment for confounders [39].

It is apparent that the quality of evidence with regards to the effectiveness of NIV in comparison to HFNOT for COVID-19 pneumonia is limited. This result is due to the broad variability in clinical practice and different guideline statements across countries and hospitals. Further large-scale studies are necessary to assess the optimal noninvasive respiratory support treatment in COVID-19. Currently, an ongoing randomized controlled trial, the RECOVERY–RS trial, aims to determine if CPAP or HFNO is effective compared to conventional oxygen therapy in reducing the mortality or/and intubation rate in COVID 19 patients [110].

## 8. Prone Position during High-Flow Nasal Oxygen or Noninvasive Ventilation in Patients with COVID-19

The favourable pathophysiological effects of prone positioning on gas exchange were depicted as early as 1974. When a patient turns from the supine to prone position, more alveolar units open as the dependent dorsal parts of the lung, which represent over 60% of the total lung mass, are more adequately ventilated, due to changes in hydrostatic pressure. Consequently, the end-expiratory lung volume may increase, ventilation distribution becomes more even, pressures are more uniformly exerted on the lungs, the ventilation–perfusion ratio improves and lung compliance increases. These effects may augment oxygenation, protect from ventilation-induced lung injury and reverse right heart failure [111]. In the last decade, the prone position was established as a therapeutic strategy in mechanically ventilated patients with severe ARDS, following the landmark PROSEVA study that demonstrated that when applied for ≥ 16 h it improves survival [112]. This finding was confirmed in subsequent meta-analyses [113,114]. 

Awake self-proning has been described in small observational, mostly retrospective cohorts in non-COVID-19 [115,116] and COVID-19 [117,118,119,120,121,122,123,124,125,126,127,128,129] patients with acute hypoxemia. Turning the awake hypoxemic patient prone was feasible, safe [117,120,125,126,130] and in most cases improved oxygenation [117,119,120,125,126,128,129], with a mean PaO_2_/FiO_2_ difference of 51.3 mmHg (95% CI 13.91–88.67) [131]. Oxygenation improvement was sustained after re-supination in patients in whom the prone position was combined with noninvasive respiratory strategies (HFNO or NIV) [117,126]. Overall, around 28% of patients eventually required invasive MV [131,132,133]. It remains inconclusive whether awake self-proning had an effect on intubation and mortality. Most investigators reported that prone sessions did not influence the intubation rates [117,121,124,125] or survival [121,124,125]. Recently, Rosén et al. conducted a multicenter randomized controlled trial in patients with COVID-19 treated with HFNO or NIV for severe hypoxemia [134]. Patients were randomly assigned to protocolized prone sessions of 16 h/day or the standard of care. The study was terminated early because the primary end-point, intubation within 30 days, did not differ between the studied groups [134]. It should be noted that only a minority of patients complied with the 16 h/day in the prone position, which is in line with the low adherence to prone reported by previous investigators [135]. Some authors have proposed dexmedetomidine as a way to increase the tolerance of prolonged prone position in awake patients with COVID-19 [136]. 

In summary, awake prone position is a supplemental strategy that may improve oxygenation in patients with noninvasive respiratory management of severe hypoxemia related to COVID-19. The exact timing to implement the prone sessions, their duration and frequency as well as failure criteria are not uniformly defined. Moreover, the clinical outcomes of awake prone positioning remain vague and further large multicenter randomized trials are needed to determine the effect on intubation rate and survival. Given its feasibility and absence of serious side effects, the prone position is proposed as an additional aid to improve oxygenation provided that the patient can tolerate it. 

## 9. Limitations

The main limitations of this narrative review are related to its nature. No peer-reviewed methodology was applied in the included studies. In addition, the literature lacks randomized controlled trials, adequately investigating the efficacy of noninvasive respiratory management in patients with severe COVID-19 hypoxemia. Instead, most studies were observational and retrospective in nature. However, we included and summarized all the published data in the field. Recommendations are based on the qualitative interpretation of available evidence, previous knowledge of the efficacy of studied respiratory modalities on hypoxemia as well as the authors´ experience.

## 10. Conclusions

In summary, available evidence are inconclusive with respect to the real effect of HFNO and NIV on outcome of patients with severe hypoxemia as a result of SARS-COV-2. Nevertheless, the pandemic wave left no place for prospective randomized controlled trials in this setting. Based on the experience prior to COVID-19 and on the few studies conducted in patients with SARS-COV-2, nearly 50% of the patients could come through without intubation, receiving only noninvasive respiratory treatment. This percentage is not negligible if one considers the ICU beds that can be spared and the ICU-related complications that can be avoided. Further large-scale trials will identify the group of COVID-19 patients in which noninvasive respiratory management is more beneficial and the risk is minimal. It is also important to collect research data that will provide evidence for the establishment of solid predictors for NIV failure. Meanwhile, there is no reason not to exploit HFNO and/or NIV when conventional oxygen strategies fail, provided that there are no indications for imminent intubation, the patient is closely monitored and precautions to avoid intubation delay and virus transmission are respected. The resulting reduction in invasive MV and ICU burden could be lifesaving. An algorithm for the safe and efficient application of HFNO and NIV is provided (Figure 1). Finally, turning the patient prone while receiving noninvasive respiratory treatment is another weapon in the armamentarium of physicians against endotracheal intubation and invasive MV of patients with severe hypoxemia due to COVID-19. 

## Figures and Tables

**Table 1:** Evidence for the use of High-Flow Nasal Oxygen in patients with COVID-19-associated hypoxemic respiratory failure.

Study	Design	No	HFNO Rate	HFNO Failure	Mortality if HFNO Fails	Other Outcomes
Wang, 2020 [8]	Retrospective MC	27	63%	41%	NA	The HFNO failure rate was 0% in patients with PaO_2_/FiO_2_ > 200 mm Hg.
Patel, 2020 [9]	Retrospective SC	445	23.3%	35.6%	34.4%	-
Demoule, 2020 [11]	Retrospective MC	379	39%	56%	25%	In a propensity scored analysis, HFNO was associated with a reduced proportion of IMV compared with no HFNO.
Xu, 2020 [12]	Retrospective MC	45	82.2%	51%	NA	-
Yang, 2020 [13]	Retrospective SC	52	63.5%	NA	NA	-
Bhatraju, 2020 [14]	Retrospective MC	24	42%	NA	NA	-
Xia, 2020 [27]	Retrospective MC	290	14.8%	46.5%	65%	Male sex and hypoxemia severity at admission were independent predictors of HFNO failure
Bonnet, 2021 [10]	Retrospective MC	138	55%	51%	16%	HFNO was compared with SOT using weighted propensity score. HFNO was associated with a lower rate of IMV. No difference in ICU LOS and mortality.
Mellado-Artigas, 2021 [28]	Prospective MC	468	41%	38%	26%	Propensity matched cohort of 122 pts showed that compared to early IMV, HFNO increased ventilator free days and reduced ICU LOS.
Chandel, 2021 [29]	Retrospective MC	272	100%	39.7%	45.4%	ROX >3.0 at 2, 6, and 12 h after HFNO was 85.3% sensitive for HFNO success. No outcomes difference between early or late (>48 h) intubation.
Liu, 2021 [30]	Retrospective MC	652	56%	56%	49%	A normogram that predicted NIRS failure on Day 1 was developed. Patients in whom NIRS fails have a high risk of death might benefit from early triage and close monitoring.
Sayan, 2021 [31]	Retrospective MC	43	55.8%	54.2%	92%	Compared to patients receiving SOT, those managed with HFNO had lower intubation rate (54.2% vs. 84.2%) and lower mortality (50% vs. 84.2%)

HFNO failure refers to escalation to noninvasive mechanical ventilation or endotracheal intubation. No = number of patients included; HFNO = High-Flow Nasal Oxygen; MC = multicenter trial; SC = single center; SOT = standard oxygen therapy; IMV = invasive mechanical ventilation; LOS = length of stay; NIRS = noninvasive respiratory strategies.

**Table 2:** Evidence for the use of NIV (CPAP or BiPAP) in patients with COVID-19-associated hypoxemic respiratory failure.

Study	Design	No	Mode	NIV Rate	Death under NIV	NIV Failure	Mortality if NIV Fails	Other Outcomes
Franco, 2020 [39]	Retrospective MC	670	CPAP BiPAP	49% 26%	22% 25%	25% 28%	32% 18%	Mortality rates using HFNO, CPAP and NIV were not significantly different, after adjusting for potential confounders
Karagiannidis, 2020 [40]	Retrospective MC	10,021	NA	3%	45%	49%	50%	NIV failure is related with mortality as high as invasive mechanical ventilation
Bertaina, 2021 [41]	Retrospective MC	1933	NA	20%	33.8%	15.9%	58.1%	Older age, hypertension, room air SatO_2_ < 92% at presentation, lymphocytopenia, and the need for antibiotic therapy during admission were independently associated with in-hospital death or intubation
Oranger, 2020 [46]	Retrospective SC	52	CPAP	73%	0%	24%	NA	-
Alviset, 2020 [42]	Retrospective SC	49	CPAP	80%	0%	62%	50%	-
Brusasco, 2021 [45]	Retrospective SC	64	CPAP	100%	6%	11%	71%	Neither PaO_2_/FIO_2_ nor lung weight were predictors of CPAP failure. CPAP avoided death or intubation in 36 out of 53 patients with PaO_2_/FIO_2_ < 150 and/or lung weight > 1.5 kg
Aliberti, 2020 [43]	Prospective MC	157	CPAP	100%	22.9%	21.7%	26.5%	-
Bellani, 2021 [47]	Prospective MC	909	CPAP BiPAP	85% 15%	22.2%	15.4%	NA	10% of COVID-19 patients were treated with NIV outside the ICUs and the overall rate of success of was 65%.
Ashish, 2020 [48]	Retrospective SC	206	CPAP	8.7%	50%	NA	NA	-
Coppadoro, 2021 [49]	Retrospective MC	306	CPAP	100%	30.4%	17.6%	40.7%	Helmet CPAP treatment is feasible for several days outside ICU
Kofod, 2021 [50]	Retrospective SC	53	CPAP	83%	43%	29%	54%	-
Avdeev, 2021 [51]	Retrospective MC	61	CPAP BiPAP	73.8% 26.2%	0%	27.9%	88.2%	NIV is feasible in patients with COVID-19 outside the ICU
Menzella, 2021 [52]	Retrospective SC	79	BiPAP	100%	25.3%	26.6%	43%	-
Paternoster, 2020 [53]	Retrospective SC	11	CPAP	100%	0%	27%	67%	-
Duca, 2020 [54]	Retrospective SC	85	CPAP BiPAP	83.5% 8.2%	54.9% 57.1%	36.6% 0%	57.7% -	-
Grieco, 2021 [55]	Prospective MC RCT	109	BiPAP	49.5%	NA	30%	NA	Treatment with helmet NIV compared with HFNO resulted in no significant difference in the number of days free of respiratory support within 28 days, among patient with COVID-19 and moderate to severe hypoxemia.
Noeman-Ahmed, 2020 [56]	Retrospective SC	52	CPAP	100%	19.2%	40.4%	38%	-

NIV failure refers to escalation to endotracheal intubation. No = number of patients included; NIV = Noninvasive Ventilation; CPAP = Continuous Positive Airway Pressure; BiPAP = Bi-level Positive Airway Pressure; HFNO = High-Flow Nasal Oxygen; MC = multicenter trial; SC = single center; ICU = Intensive Care Unit; RCT = Randomized Control Trial.
